# An explainable predictive model for anxiety symptoms risk among Chinese older adults with abdominal obesity using a machine learning and SHapley Additive exPlanations approach

**DOI:** 10.3389/fpsyt.2024.1451703

**Published:** 2024-12-10

**Authors:** Tengfei Niu, Shiwei Cao, Jingyu Cheng, Yu Zhang, Zitong Zhang, Ruiling Xue, Jingxi Ma, Qian Ran, Xiaobing Xian

**Affiliations:** ^1^ Department of Basic Courses, Chongqing Medical and Pharmaceutical College, Chongqing, China; ^2^ The Second Clinical College, Chongqing Medical University, Chongqing, China; ^3^ School of Public Health, Chongqing Medical University, Chongqing, China; ^4^ Department of Rehabilitation, Chongqing General Hospital, Chongqing, China; ^5^ Department of Neurology, Chongqing General Hospital, Chongqing, China; ^6^ Operations Management and External Communications Department, The Thirteenth People’s Hospital of Chongqing, Chongqing, China; ^7^ Operations Management and External Communications Department, Chongqing Geriatrics Hospital, Chongqing, China

**Keywords:** abdominal obesity, anxiety symptoms, XGBoost, SHAP, older adults

## Abstract

**Background:**

Early detection of anxiety symptoms can support early intervention and may help reduce the burden of disease in later life in the elderly with abdominal obesity, thereby increasing the chances of healthy aging. The objective of this research is to formulate and validate a predictive model that forecasts the probability of developing anxiety symptoms in elderly Chinese individuals with abdominal obesity.

**Method:**

This research’s model development and internal validation encompassed 2,427 participants from the 2017-2018 Study of the Chinese Longitudinal Healthy Longevity Survey (CLHLS). Forty-six variables were defined based on the Health Ecology Model (HEM) theoretical framework. Key variables were screened using LASSO regression, and the XGBoost (Extreme Gradient Boosting) model was further introduced to forecast the risk of developing anxiety symptoms in the elderly with abdominal obesity. SHapley Additive exPlanations (SHAP) was adopted to further interpret and show how the eigenvalues contributed to the model predictions.

**Results:**

A total of 240 participants (9.89%) with anxiety symptoms out of 2,427 participants were included. LASSO regression identified nine key variables: looking on the bright side, self-reported economic status, self-reported quality of life, self-reported health status, watching TV or listening to the radio, feeling energetic, feeling ashamed/regretful/guilty, feeling angry, and fresh fruits. All the evaluation indicators of the XGBoost model showed good predictive efficacy. Based on the significance of the features identified by SHAP (Model Interpretation Methodology), the feature ‘looking on the bright side’ was the most important, and the feature ‘self-reported quality of life’ was the least important. The SHAP beeswarm plot illustrated the impacts of features affected by XGBoost.

**Conclusion:**

Utilizing machine learning techniques, our predictive model can precisely evaluate the risk of anxiety symptoms among elderly individuals with abdominal obesity, facilitating the timely adoption of targeted intervention measures. The integration of XGBoost and SHAP offers transparent interpretations for customized risk forecasts.

## 1 Introduction

Abdominal obesity, alternatively referred to as central obesity, is defined by the disproportionate accumulation of adipose tissue in the abdomen, particularly around the waistline and upper torso (1). Abdominal obesity is highly prevalent among older adults globally. A global meta-analysis covering 288 studies with 13.2 million people showed that the overall prevalence of abdominal obesity was as high as 41.5% (2). In Ecuador, the prevalence of abdominal obesity was 65.9% in women, compared with 16.3% in men, among individuals aged 60 years and older (3). A survey in the United Kingdom unveiled a substantial escalation in abdominal obesity between 1993 and 2008, jumping from 19.2% to 35.7% among males and 23.8% to 43.9% among females (4). Furthermore, a recent cross-sectional study of nearly half a million participants showed that the prevalence of abdominal obesity in the Chinese population was 29.1% (5). Abdominal obesity, a global public health issue, has emerged as a significant contributor to a wide array of detrimental health conditions. Compared with general obesity, abdominal obesity, as determined by waist circumference (WC), has become a stronger predictor of obesity-related diseases (6), and it is significantly associated with adverse health outcomes such as hypertension, diabetes, metabolic syndrome, disability, frailty, and all-cause mortality (7–11).

Anxiety is a prevalent mental health disorder and is associated with a range of conditions that threaten quality of life, such as cardiovascular disease, dementia, disability, chronic pain, and autoimmune and neurodegenerative diseases (12–18). Current clinical practice and public health prevention are also increasingly concerned about the direct impact of anxiety symptoms on the health of older adults (19). Meanwhile, studies in several countries have successively reported the prevalence of anxiety symptoms in the elderly population, including 13.1% in the United States (20), 17.8% in South Africa (21), and a relatively high prevalence of 21.6% in China (22). Many studies have confirmed that abdominal obesity also correlates with psychological distress and mental disorders in the elderly, especially the occurrence of anxiety symptoms (23, 24). A national survey in the United States showed that obese patients had a 25% increased risk of developing mood and anxiety disorders compared to healthy individuals (25). However, Ran Qi et al. found that abdominal obesity may be associated with a lower prevalence of anxiety symptoms (24). This paradoxical view can be explained from a biological perspective by the fact that obesity affects biological pathways related to psychiatric disorders, including immunoinflammatory processes, oxidative stress, mitochondrial disorders, HPA axis imbalances, and neurotransmitter imbalances (26). These dysregulated pathways interact with each other and may contribute to the onset of anxiety symptoms. Therefore, the relationship between abdominal obesity and anxiety symptoms and the mechanism of action still need to be explained by further research. Fortunately, anxiety symptoms in older adults can be prevented and improved. One clinical trial showed that a targeted step-by-step nursing approach for patients halved the incidence of depression and anxiety after one year, and its effects persisted for more than one year (27). Besides, the results of one study suggested that anxiety disorders remain under-recognized and under-treated in the aging population in current society (28). Based on this current situation, developing reliable tools that can accurately predict the risk of anxiety symptoms in older adults with abdominal obesity and formulating targeted interventions in advance are particularly important for improving the well-being of older adults in their later life and reducing the burden of disease associated with aging.

To precisely forecast the risk of anxiety symptoms in the elderly with abdominal obesity, it is crucial to examine the factors that are most strongly correlated with anxiety symptoms in the elderly. Former studies have found the influencing factors of anxiety in older adults, including gender, age, marital status, physical condition, economic status, quality of life, living habits, life events, religious beliefs, social support, etc. (29–32), it can be seen that the influencing factors of anxiety symptoms are multifaceted. In order to consider the factors influencing anxiety symptoms in older adults with abdominal obesity in a comprehensive and rational way, we introduced the Health Ecology Model. The Health Ecology Model emphasizes the multiple levels of environmental and individual influences and the complexity of influencing factors. It also studies the influencing factors of diseases from five perspectives: personal characteristics, behavioral lifestyles, interpersonal networks, living/working conditions, and policy environment. It is an essential theoretical model to guide the field of public health and to solve population health problems (33).

Machine learning can process big data at high speeds and in many forms, and its application in mental health has shown excellent potential. Currently, the application of machine learning in mental health mainly focuses on four areas: detection and diagnosis, prognosis and treatment, public health prevention, and clinical management, and most of the research focuses on the detection and diagnosis of mental health conditions by machine learning (34). For example, Paolo et al. used Support Vector Machine to help predict the risk of developing dementia in mild cognitive impairment so as to achieve early diagnosis of dementia (35). Raymond et al.’s study utilized Logistic Regression, Linear Support Vector Machine, and Multilayer Perceptron models to analyze social media text data to predict depression symptoms, with the Logistic Regression model performing the best (36). Among the various mental health disorders, anxiety symptoms have received relatively little attention, and these studies have been primarily directed at adolescents or limited to patients with certain types of disorders or dysfunctions (37–39). For example, Zihan Wei et al. trained six machine learning models (Logistic Regression, Lasso Regression, Random Forest, Gradient Boosting Machine, Extreme Gradient Boosting, and Multilayer Perceptron) to predict depression and anxiety symptoms in Chinese epileptic patients and observed that the Random Forest and the Multilayer Perceptron were the ones with the better prediction performance (38). Interestingly, in previous applied machine learning research, most models focused on the accuracy of their predictions and rarely interpreted their predictions in a meaningful way. Therefore, developing an interpretable predictive model remains a challenge. Interpretability of models not only enhances model transparency and user trust, but also helps researchers to better understand the predictive mechanisms within the model, which may be more useful in guiding the model development and optimization process (40, 41). XGBoost, an optimized gradient tree boosting system that integrates multiple weak tree models to build stronger learning models, provides a machine learning technique with algorithmic innovations and hyper-parametric nonlinearities that improve model predictions while controlling the occurrence of overfitting problems (40). Extreme Gradient Boosting (XGBoost) has high computational efficiency and prediction accuracy, and is capable of automatic feature selection. When there are too many predictor variables, it can control the model complexity with built-in regularization parameters to prevent overfitting of the model. Besides, XGBoost can estimate the extent to which each feature contributes to the model, which is helpful for feature selection and model interpretation (41). Léo Grinsztajn et al. used 45 datasets from different domains for testing and concluded that tree-based models are much better than deep learning/neural networks at analyzing tabular data (42). Despite the drawbacks of complex tuning parameters and large memory footprint, XGBoost has become the algorithm of choice for many machine learning tasks due to its superior performance. For example, Jili Li et al. used XGBoost, Logistic Regression, Random Forest, and Support Vector Machine to develop a predictive model of mortality risk in patients with heart failure in the intensive care unit. They observed that XGBoost had the highest prediction performance (43). However, reliable statistical performance does not necessarily guarantee the utility of these models, and healthcare workers’ understanding of machine learning models can directly affect the application of machine learning models in clinical decision-making (44). To address this issue, the SHapley Additive exPlanation (SHAP), developed by Lundebery and Lee, may be of great use. SHAP can explain and show how feature values contribute to the prediction process and provide a dynamic view of the impact of each factor to clearly demonstrate the risk probability of a disease and the role of each feature at an individual level (45). SHAP may be a good explanation for the ‘black box’ problem of machine learning models.

In summary, this study aims to use survey data from the Chinese Longitudinal Health Longevity Survey (CLHLS) from 2017 to 2018, combined with the Health Ecology Model, to classify and extract relevant factors from five perspectives: personal characteristics, behavioral lifestyles, interpersonal networks, living/working conditions, and policy environment. Then, a machine learning model based on XGBoost is constructed to predict and analyze anxiety symptoms in elderly people with abdominal obesity. Finally, SHAP analysis is used to explain how predictive variables affect anxiety symptoms, in order to help medical staff identify high-risk populations early and take timely intervention measures to reduce the risk of anxiety in elderly people with abdominal obesity, thereby promoting healthy aging.

## 2 Materials and methods

### 2.1 Data and participants

The data employed in this research originate from the ongoing CLHLS, which commenced in 1998 and subsequently conducts follow-up surveys at intervals of 2 to 3 years (46). The CLHLS, a nationwide longitudinal study of older adults, is spearheaded by the Center for Healthy Aging and Development Research at Peking University/National Institute for Development Research. It encompasses 23 Chinese provinces, municipalities, and autonomous regions, focusing on individuals aged 65 and above. Further details regarding this survey’s sampling methodologies and data quality assurance have been comprehensively documented in another publication (47). The Biomedical Ethics Committee of Peking University authorized this project (Reference Number: IRB00001052-13074). Before participating in the baseline and follow-up surveys, all participants or their legally authorized representatives provided written consent.

**Figure 1:**
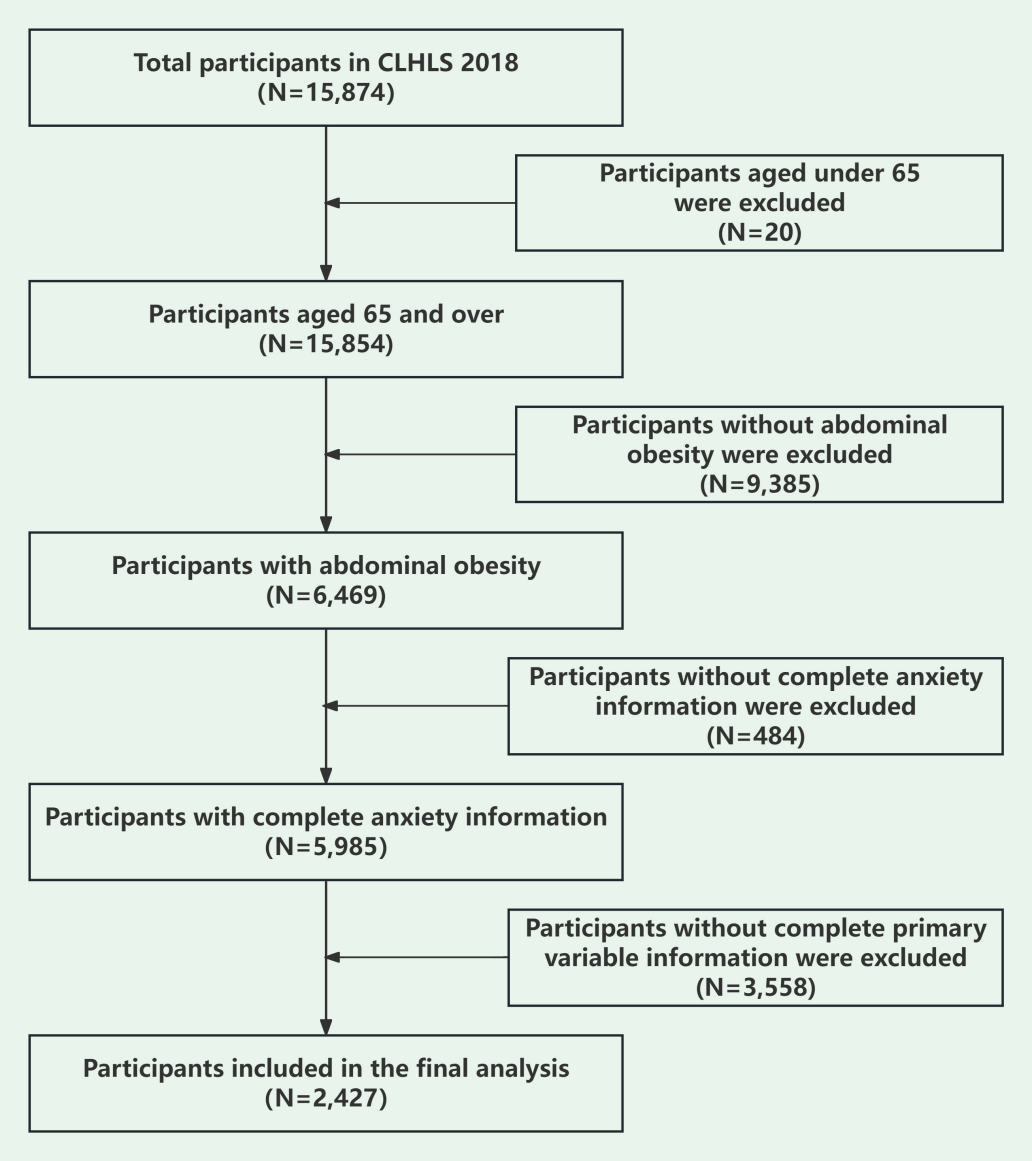
Data cleaning flow chart.

The sample size of the study was determined according to the formula for calculating sample size in cross-sectional studies[n = (Z^2^_α/2_ p q)/δ^2^] (48): (1) n denotes the sample size needed for the study; (2) p denotes the prevalence rate of anxiety symptoms in Chinese older adults; (3) q = (1-p); (4) Z_α/2_ was set at 1.96, and α was set at 0.05 for the two-sided test; and (5) δ denotes the allowed error, calculated at 0.1p. A previous study showed that the prevalence of anxiety symptoms in Chinese older adults was 21.1% (22). Based on the previous study, we calculated that a minimum of 1436 participants would be required for this study to reach the required sample size. Abdominal obesity was defined as a waist circumference greater than or equal to 90 centimeters for men and 85 centimeters for women (49). A total of 2,427 were eligible for model development and internal validation in this cross-sectional analysis. Inclusion criteria were: (1) participants aged 65 years and above; (2) participants with abdominal obesity; (3) participants with complete responses for anxiety symptoms; (4) participants who provided comprehensive responses to the main variables of screening. **Figure 1** illustrates the process of data cleaning.

### 2.2 Research variables

#### 2.2.1 Outcome variables

Anxiety symptoms were measured using the Generalized Anxiety Disorder-7 (GAD-7) scale, a self-reported scale which consists of seven dimensions based on the Diagnostic and Statistical Manual of Mental Disorders, Fourth Edition (DSM-IV) and assesses anxiety symptoms in the preceding fortnight. The GAD-7 consists of seven questions, ranging from ‘not at all’ (score = 0) to ‘almost every day’ (score = 3). The scoring scale spans from 0 to 21, with a higher aggregate score reflecting a greater severity of anxiety symptoms. Participants were considered to have anxiety symptoms when the GAD-7 score was >5 (50). The Cronbach’s coefficient for this scale stands at 0.919.

#### 2.2.2 Predictive variables

Based on the five aspects of Health Ecology Model, clinical implications and scientific knowledge of anxiety, and predictors identified in preceding publications, we considered predictors associated with the presence of anxiety symptoms in the environment on a full-cycle basis (51, 52). We screened 46 possible predictors from five perspectives: personal characteristics, behavioral lifestyles, interpersonal networks, living/working conditions, and policy environment. To be specific, they are gender, residence, age, ethnic group, co-residents, education level, occupation before retirement, economic status, marital status, smoking, drinking, exercise, insurance, Body Mass Index (BMI), Activity of Daily Living (ADL), Instrumental Activity of Daily Living (IADL), hypertension, diabetes, heart disease, stroke or cerebrovascular disease, self-reported quality of life, self-reported health status, looking on the bright side, keeping tidy and clean, feeling energetic, feeling ashamed/regretful/guilty, feeling angry, feeling busy, feeling people not trustworthy, making own decisions, staple food, fresh fruits, fresh vegetables, dietary taste, cooking oil, housework, Taichi chuan, square dance, interaction with friends, other outdoor activities, garden work, reading books or newspapers, raising domestic animals, playing cards or mahjong, watching TV or listening to the radio, social activities, traveling times (**Supplementary Table S1**).

### 2.3 Statistical analysis

#### 2.3.1 Data pre-processing

All data cleansing was performed using SPSS27.0. Utilizing the inclusion and exclusion criteria, we screened 2,427 older adults with abdominal obesity aged 65 years or older with complete information on predictive and outcome variables.

In the dataset employed for the current study, all the variables were categorized and presented in frequency and percentage, with a subsequent univariate analysis being carried out through the application of Chi-square tests. After processing the data, we allocated 70% of the data to a training set and the remaining 30% to a testing set. The training set served as the foundation for developing the model, whereas the testing set facilitated the precise calibration and optimization of the model’s parameters and evaluated its generalization capabilities.

#### 2.3.2 Model construction and evaluation

Utilizing R version 4.3.0, we carried out all analyses, considering a P-value less than 0.05 as indicative of statistical significance. The subsequent procedures for constructing and assessing the model are below: (1) The least absolute shrinkage and selection operator (LASSO) was adopted, and the key variables were selected from the 46 variables by applying 10-fold cross-validation. (2) The dataset was randomly divided into two distinct subsets: a training set and a testing set (the seed number was 123) at a ratio of 7:3, and a predictive model was developed utilizing the Extreme Gradient Boosting (XGBoost), and its performance was tested. (3) Area, sensitivity, specificity, accuracy, recall rate and F1 score under the Receiver Operating Characteristic (ROC) curve were utilized for assessing the model’s performance. Calibration curves and Hosmer-Lemeshow goodness of fit tests were adopted to compare the degree of fit between the training and test set. Decision Curve Analysis (DCA) was further introduced to evaluate the value and comparative advantages of the model in the application scenario.

#### 2.3.3 Model interpretation

To provide a deeper understanding of how each feature variable factored into the prediction, we utilized SHAP approach to bolster the interpretability of our machine-learning model. This method calculates the individual contribution value of each feature, thereby recognizing it as a contributory element to the model’s prediction (53). To explain how predictor variables can affect anxiety symptoms, we computed the average absolute value of its corresponding SHAP value. The ultimate prediction was then derived by aggregating the contribution values of all the features. Bar charts of variable contributions based on absolute SHAP value were created to show the specific significant contributions of each variable clearly. To explain in detail the prediction of anxiety symptoms in older adults with abdominal obesity, we also randomly selected a patient for an individual demonstration and visual interpretation using force plots.

## 3 Results

### 3.1 Prevalence and baseline features of anxiety symptoms among elderly individuals with abdominal obesity

The study comprised 2,427 participants, among whom 1,428 (58.84%) were women, and a total of 240 participants suffered from anxiety symptoms, with a prevalence rate of 9.89%. Of the 46 variables included in the study, gender, education years, residence, economic status, drinking, BMI, IADL, self-reported quality of life, self-reported health status, looking on the bright side, keeping things clean and tidy, feeling energetic, feeling ashamed/regretful/guilty, feeling angry, feeling busy, distrust of others, staple food, fresh fruits, vegetables, cooking oil, reading books or newspapers, garden work, playing mahjong or cards, and watching TV or listening to the radio have statistical significance (P<0.05) (**Supplementary Table S2**).

### 3.2 Identifying predictors

**Figure 2:**
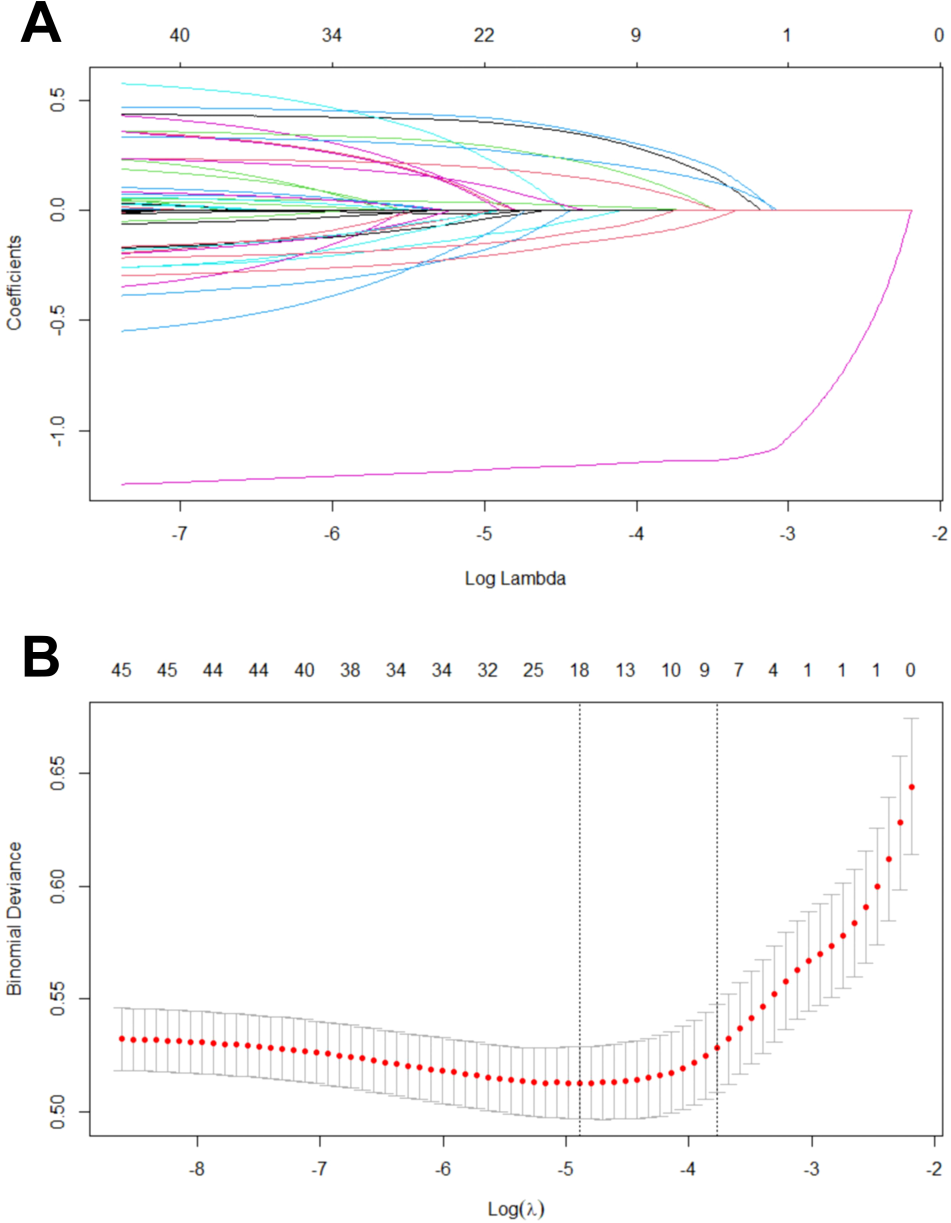
Variable screening process of Lasso regression. **(A)** Lasso coefficient curves for candidate features; **(B)** The best parameter (lambda) selected by ten-fold cross-validation, where a perpendicular dotted-line is drawn at the best value, using the minimum standard and the constraints defined by 1 standard deviation.

The results are shown in **Figure 2**, where nine key variables were screened using Lasso Regression and further incorporated into the predictive model: looking on the bright side, economic status, self-reported quality of life, self-reported health status, watching television or listening to the radio, feeling energetic, feeling ashamed/regretful/guilty, feeling angry, and fresh fruits.

### 3.3 Performance of the XGBoost model

**Figure 3:**
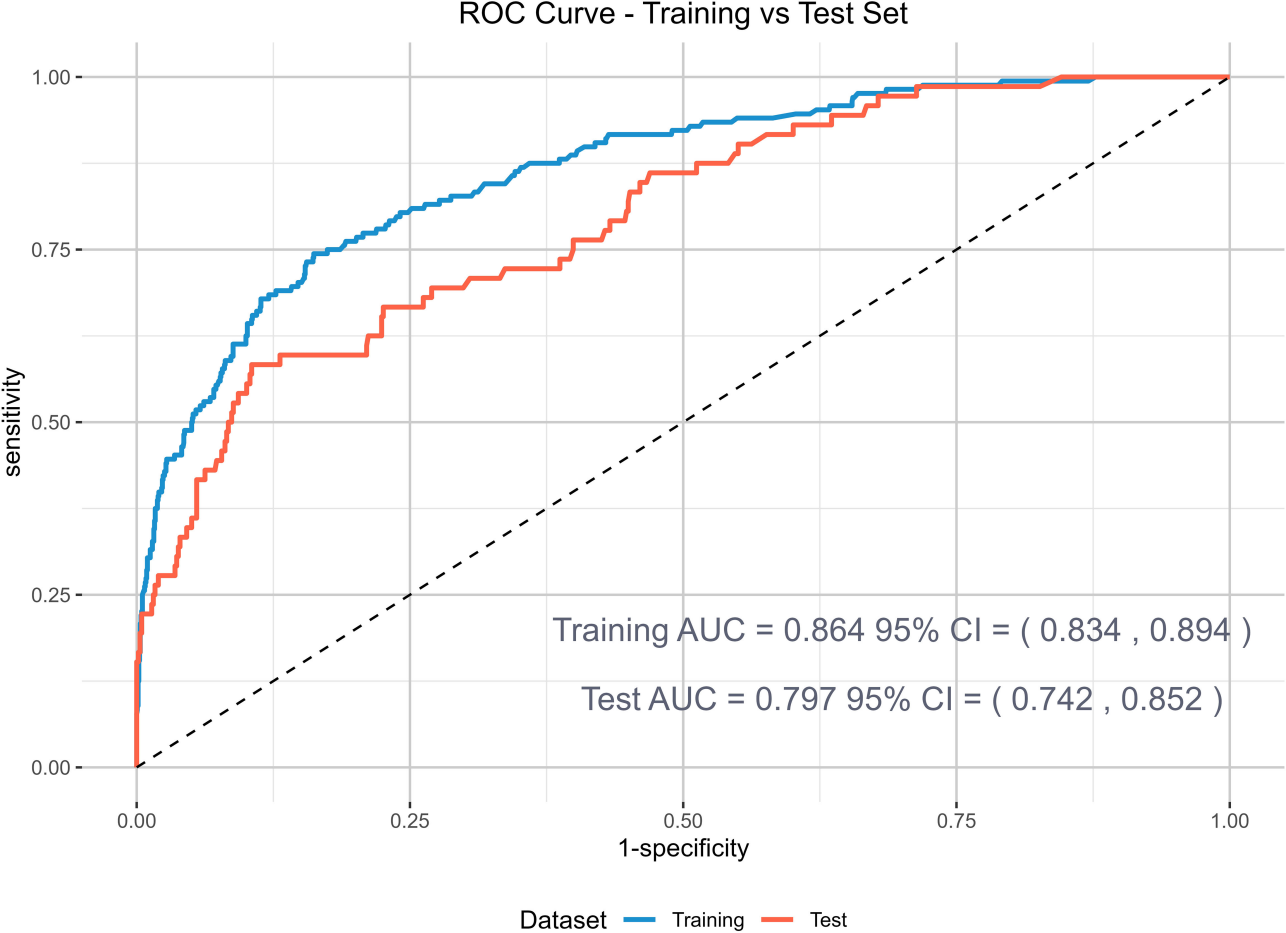
XGBoost ROC curves generated from the training and test datasets.

The ROC curve in **Figure 3** summarizes the detection and prediction performance of anxiety symptoms in older adults with abdominal obesity. The ROC curve shows a functional relationship between sensitivity (proportion of anxiety-positive cases receiving a positive marker for anxiety symptoms) and 1-specificity (proportion of anxiety-negative cases receiving a positive marker for anxiety symptoms). The results suggested that the XGBoost model displayed a favorable area under the ROC curve, both in the training set and the test set, which were 0.868 [95%CI: 0.838-0.897] and 0.793 [95%CI: 0.738-0.848] respectively. **Table 1** summarizes the sensitivity, specificity, accuracy, recall rate and F1 score of the model training and test sets. The model had a good prediction effect.

**Table 1:** Model performance evaluation metrics.

	AUC	Accuracy	Recall	Specificity	F1 Score	Sensitivity
Training set	0.868	0.921	0.855	0.928	0.723	0.855
Test set	0.793	0.911	0.834	0.923	0.713	0.834

AUC, Area Under the Curve.

**Figure 4:**
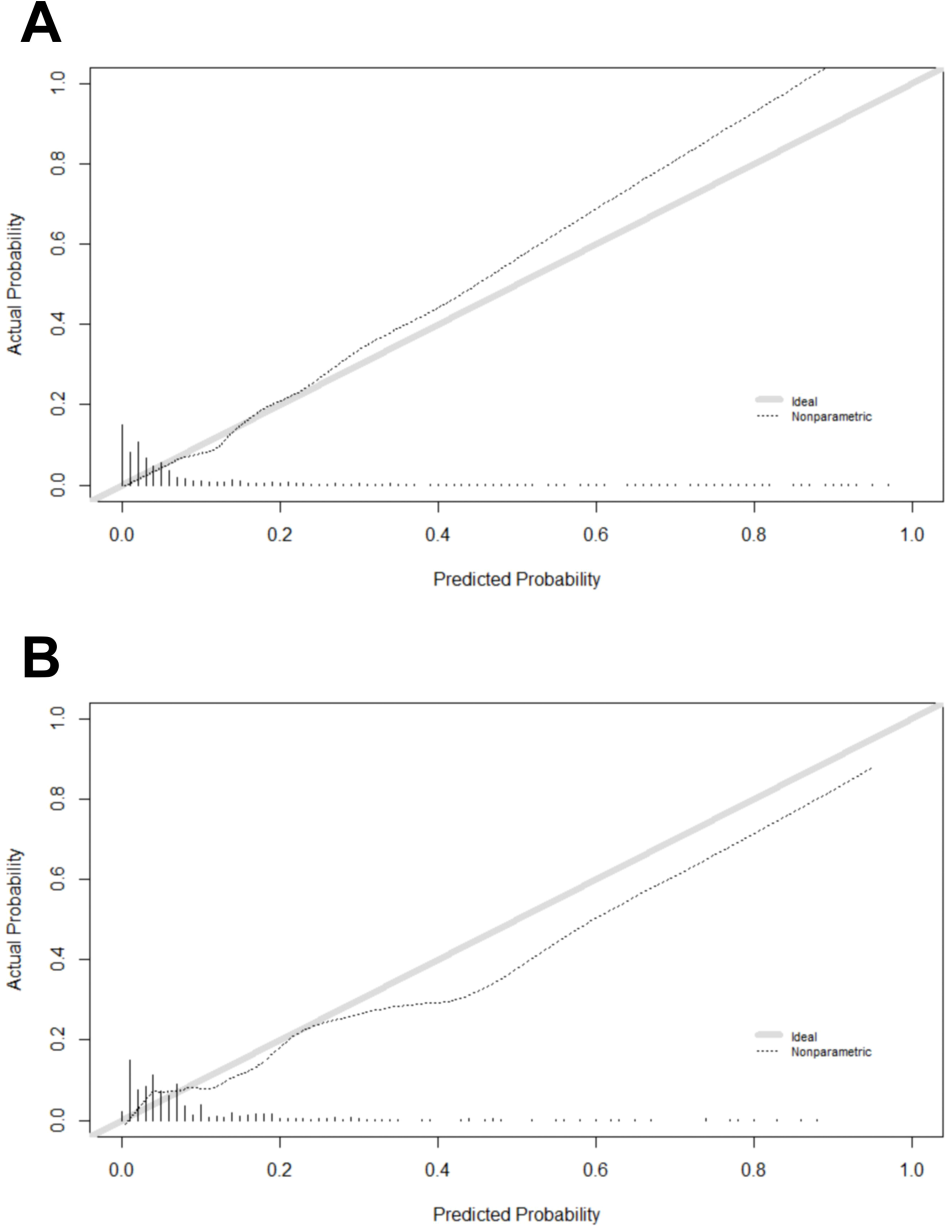
**(A)** Calibration plot for the training dataset. **(B)** Calibration plot for the test dataset.

The evaluation of the XGBoost model involved the utilization of the calibration curve and the Hosmer-Lemeshow goodness of fit test, where a P-value greater than 0.05 signified an excellent fit for the model. The test results demonstrated a satisfactory fit for the model on the training set (χ² = 5.2955, p = 0.7081) and the test set (χ² = 2.2126, p = 0.3309). As depicted in **Figures 4A, B** (**Figure 4**), there is a high degree of equilibrium between the model’s predicted and actual probability.

**Figure 5:**
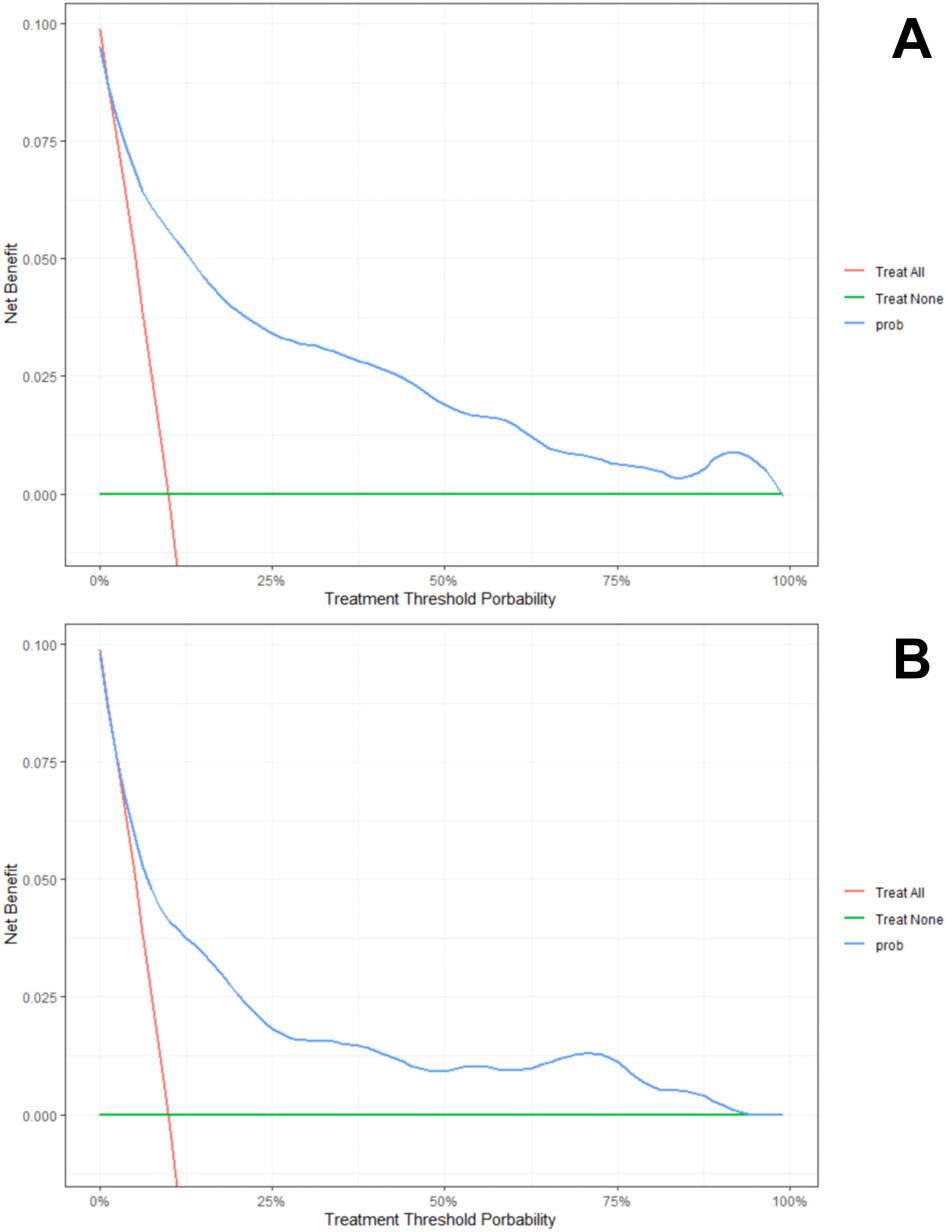
**(A)** DCA curves for the training dataset. **(B)** DCA curves for the test dataset.

The DCA method was utilized to evaluate the model’s clinical efficacy, and the results are presented in **Figures 5A, B** (**Figure 5**). As seen in the decision curve, the net benefit offered by the prediction model surpasses the net benefit of both extreme cases by a significant margin, and the threshold probability of the XGBoost model at different time points has a significant net benefit, indicating that the model has potential clinical benefit.

### 3.4 SHapley Additive exPlanations

**Figure 6:**
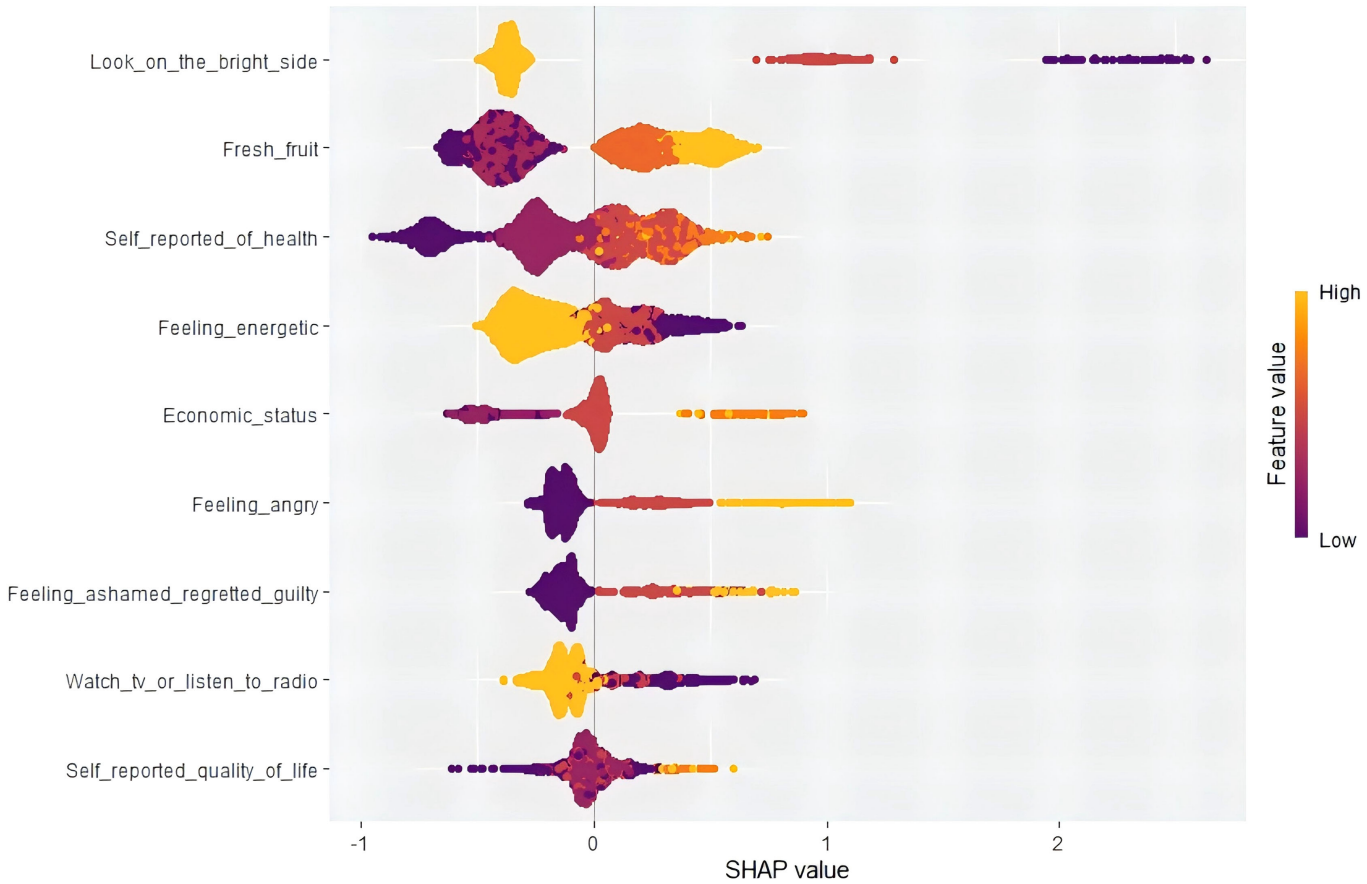
SHapley Additive exPlanation (SHAP) values.

We calculated the average SHAP value of the XGBoost model to identify important predictor variables as well as to explain their impact on the risk of developing anxiety symptoms in older Chinese adults with abdominal obesity. SHAP values served as a means to illustrate the degree to which each feature contributes to individual predictions, revealing model black-box problems. **Figure 6** shows the effect of nine features on all patients, where each dot stands for the effect of the feature on the sample.

The horizontal axis, also known as the X-axis, is a crucial element in understanding the SHAP value. This value signifies the average marginal impact that a specific feature’s value has on the model’s output across various potential combinations. It’s important to note that a SHAP value falling below zero indicates a negative contribution, suggesting that the feature detracts from the prediction. A value of zero indicates neutrality, implying no impact on the prediction. Conversely, a positive SHAP value denotes a positive contribution, indicating that the feature holds significant importance in determining the final prediction. This understanding of the SHAP value is key, as it allows us to discern the most crucial features, which will contribute positively, and the least relevant features, which may contribute negatively.

**Figure 7:**
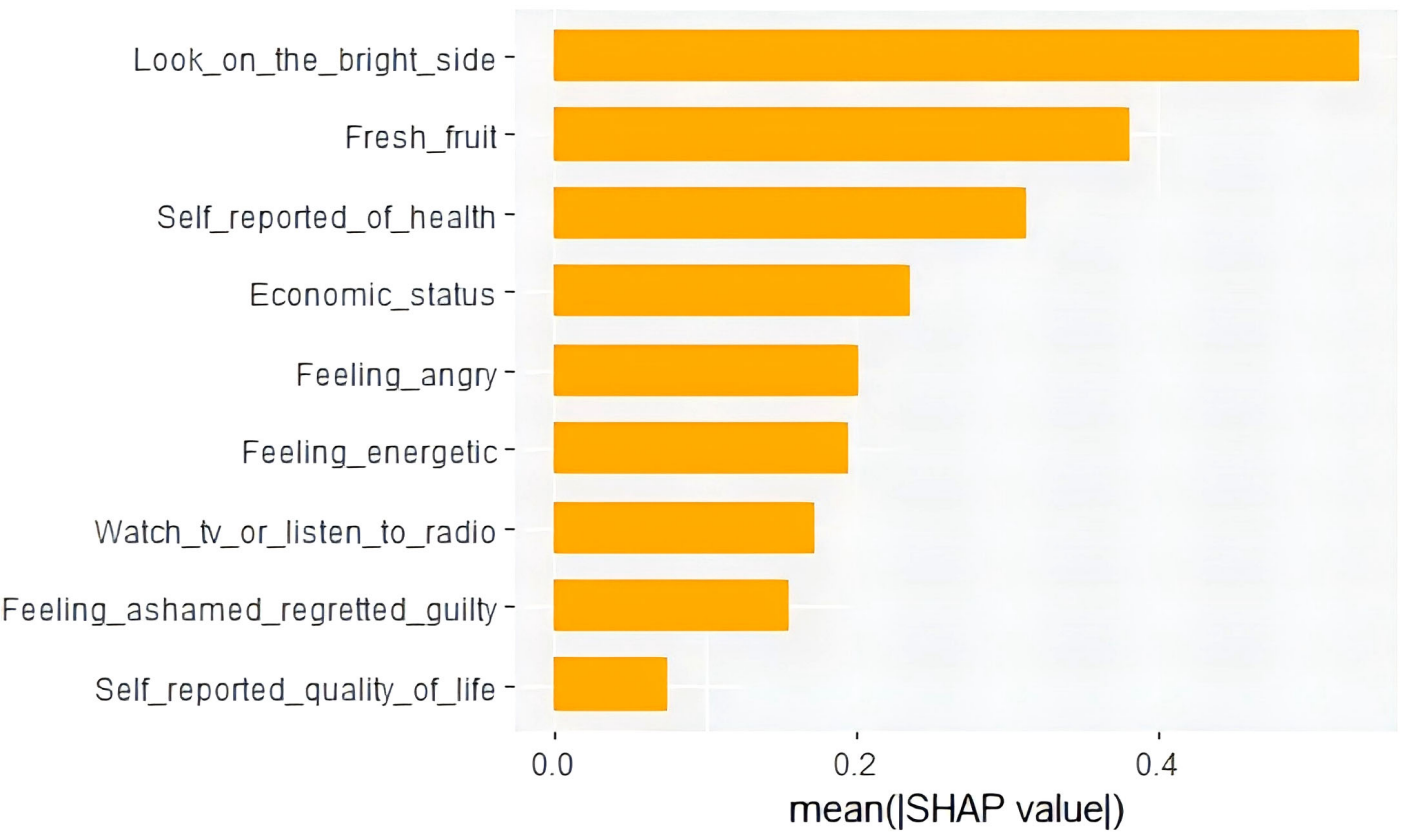
Bar chart of variable contributions based on absolute values of SHAP.

As depicted in **Figure 6**, the features at the top exhibit a more significant influence on the model’s prediction, and it is evident that each feature has been ranked based on its significance. ‘Looking on the bright side’ was the most important feature, while ‘self-reported quality of life’ was the least important feature. The vertical axis (Y-axis) comprises both left and right coordinates. On the left vertical axis, features are displayed in descending order of significance, while the right vertical axis portrays the values of these features from low to high. The color indicates the level of a feature’s contribution to the prediction, with yellow representing a high contribution and purple indicating a low contribution. As can be seen from **Figure 6**, high levels of optimism have a strong positive effect on predicting the risk of developing anxiety symptoms in older adults with abdominal obesity. The ordering of each variable according to the degree of contribution of SHAP value is shown in **Figure 7**, and it can be seen that ‘looking on the bright side’ has the highest degree of importance.

**Figure 8:**
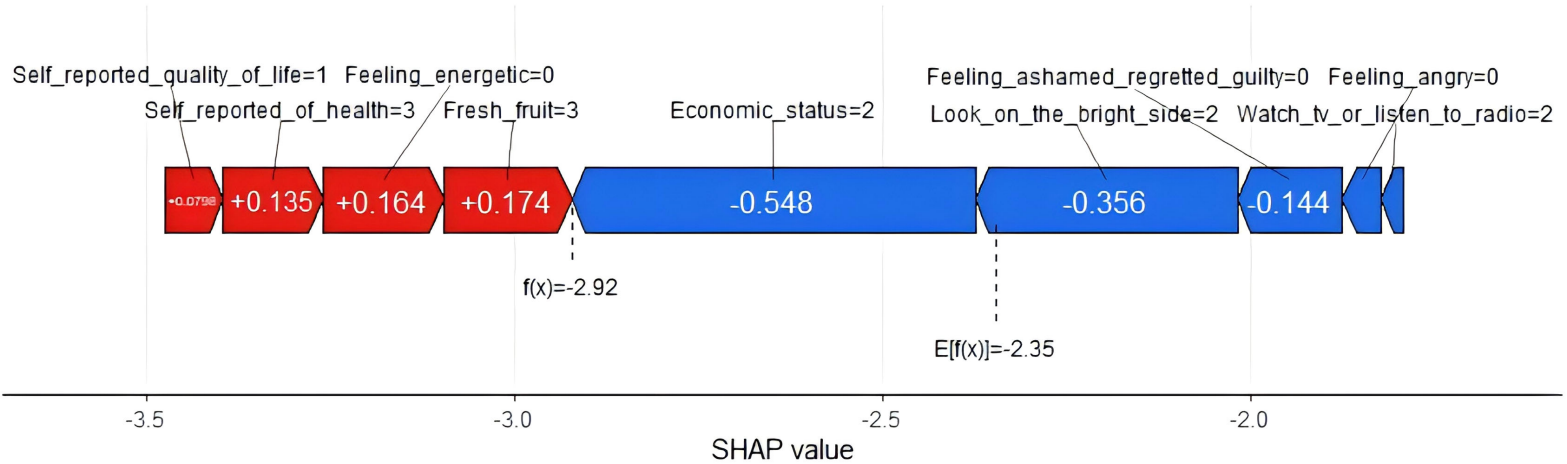
Individual prediction of anxiety symptoms in patients with abdominal obesity number 2.

To elucidate the forecast of anxiety symptoms in the elderly with abdominal obesity in detail, we used force plots to demonstrate and visually interpret the model prediction separately, as shown in **Figure 8**. The force plot presents a forecasted outcome for a randomly selected patient (patient 2). The function f(x) represents the outcome produced by the model, specifically the predicted probability for a given patient. At the same time, the base value serves as a reference point, being the average of all the model’s predictions. Features with increased predicted values are shown in red, and features with decreased predicted values are shown in blue. In addition, the red feature is the right arrow, and the blue feature is the left arrow. The size of the arrow represents the impact of the feature. In **Figure 8**, we can see that patient 2 has a lower probability of developing anxiety symptoms because some risk factors reduce the predicted outcome, such as eating fresh fruits, feeling energetic, and self-reported health status. The risk factor (feature) shifts the prediction from the base value (-2.35) to the model output (-2.92), which shows that the probability of developing anxiety symptoms is lower.

## 4 Discussion

A predictive model was constructed to evaluate the probability of developing anxiety symptoms in Chinese elderly individuals with abdominal obesity aged 65 years and above. We applied the LASSO technique to identify significant features, harnessed the predictive capabilities of the XGBoost machine learning algorithm, and concluded by constructing and validating a robust model that relied on 9 crucial features (looking on the bright side, self-reported economic status, self-reported quality of life, self-reported health status, watching TV or listening to the radio, feeling energetic, feeling ashamed/regretful/guilty, feeling angry, and fresh fruits). The model’s predictive efficacy was tested by area under the ROC curve, accuracy, recall, sensitivity, F1 score, specificity and calibration curve, and clinical decision curve. The importance of the features was determined by SHAP analysis. The results of the SHAP analyses demonstrated that ‘looking on the bright side’ was the most contributing feature, while ‘self-reported quality of life’ had the relatively lowest contribution. In addition, a random sample was selected for local interpretation of SHAP.

We found that previous studies reported a prevalence of anxiety symptoms of 21.6% in older adults (22), whereas the results of the present study showed that this figure in our abdominally obese older adults was only 9.89%. Although numerous prior investigations have delved into the correlation between abdominal obesity and mental disorders, the findings have been somewhat inconclusive. Nonetheless, some studies have revealed that abdominal obesity is associated with an increased risk of developing symptoms of depression and anxiety (54, 55). In contrast, another study conducted in a Chinese elderly population showed a protective effect of abdominal obesity on anxiety symptoms (24). This may suggest to us that the specific relationship and mechanisms regarding the relationship between abdominal obesity and anxiety symptoms deserve to be revealed in the future by designing more sophisticated experiments.

The choice of features is crucial for the development of reliable predictive models (56). The LASSO algorithm helped us to identify the 9 most important variables from the initial 46. The identification of these variables were consistent with previous studies regarding the influence of lifestyle behaviors and personality-emotional traits on anxiety symptoms in older adults, suggesting the reliability of the predictors screened by Lasso regression (57, 58). For example, our findings revealed that people who were not optimistic were more likely to experience anxiety symptoms. Similarly, previous research had demonstrated that optimism buffered the relationship between disease burden and anxiety symptoms in older adults, and that pessimism exacerbated this relationship (59). Meanwhile, some researchers have scanned relevant areas of the brain with imaging equipment and found that decreased optimism is specific to generalized anxiety disorder (60). All of these studies mentioned above helped to explain the strong potency of optimism in predicting anxiety symptoms in older adults with abdominal obesity. We also found that consumption of fresh fruits was a strong predictor of anxiety symptoms in older adults with abdominal obesity. Our study is consistent with the findings of Redzo Mujcic et al., who showed that eating fruits and vegetables may help prevent the risk of depression and anxiety disorders (61). Consumption of fresh fruits and vegetables has also been shown to be negatively associated with the occurrence of anxiety symptoms in studies of American, Canadian, and Iranian populations (62–64). In addition, a study conducted on 1,707 participants showed that the most common health risks in the structured health risk assessment were inadequate fruit/vegetable intake (84.5% of the total) and overweight/obesity (79.6% of the total) (65). These studies suggested that appropriate interventions should be implemented in primary health care to increase citizens’ awareness of healthy living and prevent the occurrence of diseases. Health self-assessment, as one of the predictors, has also been shown to be significantly associated with mental health in the elderly (66, 67). Besides, we found that feeling energetic, economic status, feeling angry, feeling ashamed/regretful/guilty, watching TV or listening to the radio, and self-reported quality of life had a potential predictive value for the risk of developing anxiety symptoms in older adults with abdominal obesity, but as can be seen from the results of the SHAP, the value of the contribution of these variables was relatively low. It is worth mentioning that in the development of our prediction model, we paid special attention to the simplicity of the measurement of the predictor in the real world, and that simple and easily accessible variables can increase the value of the model for practical applications.

We further constructed an XGBoost-based machine learning prediction model and observed a better area under the ROC curve in both the training and test sets, and the calibration curves showed a good balance between the model’s predicted probability and the actual probability. By applying the results of the model, physicians can identify individuals at high risk for anxiety symptoms among older adults with abdominal obesity, provide timely mental health assessment and intervention, and reduce the prevalence of anxiety symptoms through primary prevention. Furthermore, we also performed a decision curve analysis to provide more flexible options for providers in different domains. In particular, doctors, nurses, healthcare workers, and community workers can determine suitable thresholds based on their specialized domain knowledge to get the best net benefit. For instance, they can dynamically adjust the threshold of the machine learning model based on the prevalence level of anxiety symptoms in older adults with abdominal obesity in the region or the ability of the local health organization to recognize anxiety symptoms. If the region has a higher prevalence of anxiety symptoms, they can appropriately increase the threshold of the machine learning model to achieve the highest net benefit. Psychiatric hospitals possess a higher ability to recognize anxiety symptoms than other low-level hospitals, so doctors in these psychiatric hospitals could appropriately increase the threshold level when applying the results of our model. In addition to the above applications, the development of this prediction model suggests the possibility of using non-clinical data to predict the occurrence of anxiety symptoms in elderly people with abdominal obesity, and researchers can use this model to conduct larger epidemiological studies to explore the causal relationship between abdominal obesity and anxiety, and promote scientific research progress in related fields.

Traditional machine learning algorithms are often criticized by researchers for their lack of transparency and interpretability. In order to better understand the internal logic and decision rules behind the model predictions, one of the strengths of this study is the use of the SHAP method to explain machine learning models and reveal the ‘black box’ problem of machine learning models. In the final results, we can clearly observe the degree of contribution of each variable, for example, we found that “looking on the bright side” was the most valuable predictor of developing anxiety symptoms in older adults with abdominal obesity. The impact of each factor on a randomly selected patient can be seen in the SHAP force plot, in which eating fruits, feeling energetic, and self-reported good health reduced the risk of developing anxiety symptoms. With the help of the SHAP technique, we can focus on the predictors that lead to anxiety symptoms based on the individual level and understand the individualized performance of each factor’s contribution, thus providing the right guidance for the subsequent formulation of personalized preventive interventions. In the case of the patient shown in the force plots of SHAP, despite the low risk of anxiety symptoms in this patient, we can further reduce the patient’s risk of anxiety symptoms by psychological counseling and developing personalized lifestyle interventions, such as increasing fresh fruits intake, adjusting outdoor interactions, and enhancing health education.

Overall, the SHAP used in this study provides a way to unlock the black box of the machine learning model, which allows us to better understand the results of the XGBoost model’s prediction of the risk of developing anxiety symptoms in Chinese older adults with abdominal obesity. In addition to helping us in the early identification of anxiety symptoms in older adults with abdominal obesity, the results of this study can also help us to develop individual intervention strategies through the interpretability of SHAP.

## 5 Limitations

This study has several limitations. First, although these data are from a nationally representative survey, the unique inclusion criteria for older adults with abdominal obesity in this study excluded a large number of participants, and the representativeness of the data may have been compromised. Although we performed calculations of the minimum sample size required for the study and found that the sample size for this study was much larger than the minimum sample size required, further big data validation studies are still necessary. Second, our choice of variables was limited by the content of the database questionnaire, and therefore there is no guarantee that all potential influences were included in this study. Third, although our data processing methods have been validated in a large number of previous studies, the categorization of variables and the different criteria for classifying them may still have some impact on the results. Fourth, the predictors in our study were all measured by self-reported questions, which may lead to some information bias. Fifth, this study was a cross-sectional study and therefore the results obtained did not support causal inferences between variables. Finally, although certain methods were used to ensure the reliability and generalizability of the model, the actual results still need to be validated in an external and independent population.

## 6 Conclusion

In conclusion, we successfully utilized a machine learning approach to identify anxiety symptoms in Chinese older adults with abdominal obesity. The XGBoost model exhibited remarkable proficiency in this investigation, and the tandem of XGBoost and SHAP offered a transparent explanation for personalized risk forecasts. High-performance modelling is valuable for early identification and intervention in older adults with abdominal obesity who are potentially at risk of developing anxiety symptoms. This can help to improve the subsequent emotional state and quality of life of older adults, increase their well-being in later life, reduce the burden of disease among them, and contribute to the goal of healthy aging.

## Data Availability

Publicly available datasets were analyzed in this study. This data can be found here: the Chinese Longitudinal Healthy Longevity Survey (CLHLS), https://doi.org/10.18170/DVN/WBO7LK.
