# Supplementary material for: An explainable predictive model for anxiety symptoms risk among Chinese older adults with abdominal obesity using a machine learning and SHapley Additive exPlanations approach
Source: Front Psychiatry. 2024 Dec 10;15:1451703. doi: 10.3389/fpsyt.2024.1451703 (PMC11666561; doi:10.3389/fpsyt.2024.1451703)
Supplement: Supplementary file 1 [file Table1.docx]

Supplementary Material

# Supplementary Table S1. Questionnaire Items and Classification Criteria of Covariates.

| **Variables** | **Questionnaire Items** | **Classification Criteria** |
| --- | --- | --- |
| Gender | Gender | 0=Female |
|  |  | 1=Male |
| Residence | Current residence area of interviewee | 0=Rural |
|  |  | 1=Urban |
| Age | Current Age | 0=65-70 |
|  |  | 1=71-80 |
|  |  | 2=81-90 |
|  |  | 3=>90 |
| Ethnic group | Ethnic group | 0=Other |
|  |  | 1=Han |
| Co-residents | Co-residents | 0=Living with family |
|  |  | 1=Alone |
|  |  | 2=In a nursing home |
| Education level | How many years did you attend school? | 0=0 years |
|  |  | 1=1-6 years |
|  |  | 2=>6 years |
| Occupation before retirement | What was your primary occupation before age 60? | 0=Non-manual labor |
|  |  | 1=Manual labor |
| Economic status | How do you rate your economic status compared with others in your local area? | 0=Very rich |
|  |  | 1=Rich |
|  |  | 2=So so |
|  |  | 3=Poor |
|  |  | 4=Very poor |
| Marital status | Current marital status | 0=Married |
|  |  | 1=Divorced/Widowed/Never married |
| Smoking | Do you smoke at the present time? | 0=No |
|  |  | 1=Yes |
| Drinking | Do you drink alcohol at the present time? | 0=No |
|  |  | 1=Yes |
| Exercise | Do you do exercises regularly at present? | 0=No |
|  |  | 1=Yes |
| Insurance | Do you participated in public old age insurance? | 0=No |
|  |  | 1=Yes |
| Body Mass Index(BMI) | Weight/Height, measured directly | 0=<18.5 |
|  |  | 1=18.5-23.9 |
|  |  | 2=23.9-27.9 |
|  |  | 3=≥28 |
| Activity of Daily Living (ADL) ^a^ | Have you been limited in bathing because of a health problem?  Have you been limited in dressing because of a health problem?  Have you been limited in toilet because of a health problem?  Have you been limited in indoor transfer because of a health problem?  Have you been limited in continence because of a health problem?  Have you been limited in eating because of a health problem? | 0=No |
|  |  | 1=Yes |
| Instrumental Activity of Daily Living (IADL) ^b^ | Can you visit your neighbors by yourself?  Can you go shopping by yourself?  Can you cook a meal by yourself whenever necessary?  Can you wash clothing by yourself whenever necessary?  Can you walk continuously for 1 kilometer at a time by yourself?  Can you lift a weight of 5kg, such as a heavy bag of groceries?  Can you continuously crouch and stand up three times?  Can you take public transportation by yourself? | 0=No |
|  |  | 1=Yes |
| Hypertension | Are you suffering from hypertension? | 0=No |
|  |  | 1=Yes |
| Diabetes | Are you suffering from diabetes? | 0=No |
|  |  | 1=Yes |
| Heart disease | Are you suffering from heart disease? | 0=No |
|  |  | 1=Yes |
| Stroke or cerebrovascular disease | Are you suffering from stroke or cerebrovascular disease? | 0=No |
|  |  | 1=Yes |
| Self-reported quality of life | How do you rate your life at present? | 0=Very good |
|  |  | 1=Good |
|  |  | 2=So so |
|  |  | 3=Bad |
|  |  | 4=Very bad |
| Self-reported health status | How do you rate your health at present? | 0=Very good |
|  |  | 1=Good |
|  |  | 2=So so |
|  |  | 3=Bad |
|  |  | 4=Very bad |
| Looking on the bright side | Do you always look on the bright side of things? | 0=Always/Often |
|  |  | 1=Sometimes |
|  |  | 2=Seldom/Never |
| Keeping tidy and clean | Do you like to keep your belongings neat and clean? | 0=Always/Often |
|  |  | 1=Sometimes |
|  |  | 2=Seldom/Never |
| Feeling energetic | Do you feel energetic? | 0=Always/Often |
|  |  | 1=Sometimes |
|  |  | 2=Seldom/Never |
| Feeling ashamed/regretful/guilty | Have you been ashamed, regretful, or felt guilty about things you've done? | 0=Always/Often |
|  |  | 1=Sometimes |
|  |  | 2=Seldom/Never |
| Feeling angry | Are you angry at people or things you don't like around you? | 0=Always/Often |
|  |  | 1=Sometimes |
|  |  | 2=Seldom/Never |
| Feeling busy | Do you feel busy? | 0=No |
|  |  | 1=Yes |
| Feeling people not trustworthy | Do you feel that people around you are not trustworthy? | 0=Always/Often |
|  |  | 1=Sometimes |
|  |  | 2=Seldom/Never |
| Making own decisions | Can you make your own decisions concerning your personal affairs? | 0=Always/Often |
|  |  | 1=Sometimes |
|  |  | 2=Seldom/Never |
| Staple food | Please tell us the staple food you eat. | 0=Rice |
|  |  | 1=Corn |
|  |  | 2=Wheat |
|  |  | 3=Half rice and half wheat |
|  |  | 4=Other |
| Fresh fruit | Do you eat fresh fruit? | 0=Everyday/Almost everyday |
|  |  | 1=Quite often |
|  |  | 2=Occasionally |
|  |  | 3=Rarely/Never |
| Fresh vegetables | Do you eat fresh vegetables? | 0=Everyday/Almost everyday |
|  |  | 1=Quite often |
|  |  | 2=Occasionally |
|  |  | 3=Rarely/Never |
| Dietary taste | What kind of flavor do you mainly have? | 0=Insipidity |
|  |  | 1=Salty |
|  |  | 2=Sweet |
|  |  | 3=Hot |
|  |  | 4=Other |
| Cooking oil | What kind of grease do you mainly use for cooking? | 0=Vegetable grease/gingili grease |
|  |  | 1=Animal’s fat |
| Housework | Do you now perform the following activities regularly?  -Housework | 0=Almost/At least once a week |
|  |  | 1=At least once a month |
|  |  | 2=Sometimes/Never |
| Taichi chuan | Do you now perform the following activities regularly?  -Tai chi chuan | 0=Almost/At least once a week |
|  |  | 1=At least once a month |
|  |  | 2=Sometimes/Never |
| Square dance | Do you now perform the following activities regularly?  -Square dance | 0=Almost/At least once a week |
|  |  | 1=At least once a month |
|  |  | 2=Sometimes/Never |
| Interaction with friends | Do you now perform the following activities regularly?  -Interaction with friends | 0=Almost/At least once a week |
|  |  | 1=At least once a month |
|  |  | 2=Sometimes/Never |
| Other outdoor activities | Do you now perform the following activities regularly?  -Other outdoor activities | 0=Almost/At least once a week |
|  |  | 1=At least once a month |
|  |  | 2=Sometimes/Never |
| Garden work | Do you now perform the following activities regularly?  -Garden work | 0=Almost/At least once a week |
|  |  | 1=At least once a month |
|  |  | 2=Sometimes/Never |
| Reading books or newspapers | Do you now perform the following activities regularly?  -Read newspapers/books | 0=Almost/At least once a week |
|  |  | 1=At least once a month |
|  |  | 2=Sometimes/Never |
| Raising domestic animals | Do you now perform the following activities regularly?  -Raise domestic animals | 0=Almost/At least once a week |
|  |  | 1=At least once a month |
|  |  | 2=Sometimes/Never |
| Playing cards or mahjong | Do you now perform the following activities regularly?  -Play cards or mahjong | 0=Almost/At least once a week |
|  |  | 1=At least once a month |
|  |  | 2=Sometimes/Never |
| Watching TV or listening to the radio | Do you now perform the following activities regularly?  -Watch TV and listen to radio | 0=Almost/At least once a week |
|  |  | 1=At least once a month |
|  |  | 2=Sometimes/Never |
| Social activities | Do you now perform the following activities regularly?  -Social activities | 0=Almost/At least once a week |
|  |  | 1=At least once a month |
|  |  | 2=Sometimes/Never |
| Traveling times | How many tours beyond home city/county have you made in the past two years? | 0=0 |
|  |  | 1=1 |
|  |  | 2=≥2 |

Note: a: If participants were able to complete the six items without assistance, they did not have an ADL disability; otherwise, they had an ADL disability.

b: If participants were able to complete the eight items without assistance, they did not have an IADL disability; otherwise, they had an IADL disability.

**Supplementary Table S2 Description of basic characteristics**

| **Variables** | **Total**  **(n = 2,427)** | **Without anxiety**  **(n = 2,187)** | **Anxiety**  **(n = 240)** | **Statistic** | **P** |
| --- | --- | --- | --- | --- | --- |
|  |  |  |  |  |  |
| **Gender, n (%)** |  |  |  | χ²=15.82 | <.001 |
| Female | 1,428 (58.84) | 1,258 (57.52) | 170 (70.83) |  |  |
| Male | 999 (41.16) | 929 (42.48) | 70 (29.17) |  |  |
| **Residence, n (%)** |  |  |  | χ²=0.23 | 0.630 |
| Rural | 956 (39.39) | 858 (39.23) | 98 (40.83) |  |  |
| Urban | 1,471 (60.61) | 1,329 (60.77) | 142 (59.17) |  |  |
| **Ethnic group, n (%)** |  |  |  | χ²=0.77 | 0.381 |
| Other | 87 (3.58) | 76 (3.48) | 11 (4.58) |  |  |
| Han | 2,340 (96.42) | 2,111 (96.52) | 229 (95.42) |  |  |
| **Co-resident**s**, n (%)** |  |  |  | χ²=2.75 | 0.253 |
| Living with family | 1,956 (80.59) | 1,772 (81.02) | 184 (76.67) |  |  |
| Living alone | 388 (15.99) | 341 (15.59) | 47 (19.58) |  |  |
| Living in a nursing home | 83 (3.42) | 74 (3.38) | 9 (3.75) |  |  |
| **Education years, n (%)** |  |  |  | χ²=15.42 | <.001 |
| 0 years | 929 (38.28) | 812 (37.13) | 117 (48.75) |  |  |
| 1-6 years | 890 (36.67) | 807 (36.90) | 83 (34.58) |  |  |
| >6 years | 608 (25.05) | 568 (25.97) | 40 (16.67) |  |  |
| **Occupation before retirement, n (%)** |  |  |  | χ²=7.02 | 0.008 |
| Non-manual worker | 515 (21.22) | 480 (21.95) | 35 (14.58) |  |  |
| Manual worker | 1,912 (78.78) | 1,707 (78.05) | 205 (85.42) |  |  |
| **Economic status, n (%)** |  |  |  | χ²=99.02 | <.001 |
| Very rich | 97 (4.00) | 95 (4.34) | 2 (0.83) |  |  |
| Rich | 508 (20.93) | 488 (22.31) | 20 (8.33) |  |  |
| So so | 1,634 (67.33) | 1,469 (67.17) | 165 (68.75) |  |  |
| Poor | 162 (6.67) | 118 (5.40) | 44 (18.33) |  |  |
| Very poor | 26 (1.07) | 17 (0.78) | 9 (3.75) |  |  |
| **Marital status, n (%)** |  |  |  | χ²=0.87 | 0.350 |
| Married | 1,293 (53.28) | 1,172 (53.59) | 121 (50.42) |  |  |
| Divorced/Widowed/Never married | 1,134 (46.72) | 1,015 (46.41) | 119 (49.58) |  |  |
| **Smoking, n (%)** |  |  |  | χ²=1.55 | 0.213 |
| No | 2,112 (87.02) | 1,897 (86.74) | 215 (89.58) |  |  |
| Yes | 315 (12.98) | 290 (13.26) | 25 (10.42) |  |  |
| **Drinking, n (%)** |  |  |  | χ²=6.86 | 0.009 |
| No | 2,042 (84.14) | 1,826 (83.49) | 216 (90.00) |  |  |
| Yes | 385 (15.86) | 361 (16.51) | 24 (10.00) |  |  |
| **Exercise, n (%)** |  |  |  | χ²=1.83 | 0.176 |
| No | 1,387 (57.15) | 1,240 (56.70) | 147 (61.25) |  |  |
| Yes | 1,040 (42.85) | 947 (43.30) | 93 (38.75) |  |  |
| **Insurance, n (%)** |  |  |  | χ²=0.08 | 0.774 |
| No | 204 (8.41) | 185 (8.46) | 19 (7.92) |  |  |
| Yes | 2,223 (91.59) | 2,002 (91.54) | 221 (92.08) |  |  |
| **BMI ^a^, n (%)** |  |  |  | χ²=9.80 | 0.020 |
| <18.5 | 73 (3.01) | 60 (2.74) | 13 (5.42) |  |  |
| 18.5-23.9 | 878 (36.18) | 780 (35.67) | 98 (40.83) |  |  |
| 23.9-27.9 | 1,051 (43.30) | 964 (44.08) | 87 (36.25) |  |  |
| ≥28 | 425 (17.51) | 383 (17.51) | 42 (17.50) |  |  |
| **ADL ^b^, n (%)** |  |  |  | χ²=0.01 | 0.940 |
| No | 2,077 (85.58) | 1,872 (85.60) | 205 (85.42) |  |  |
| Yes | 350 (14.42) | 315 (14.40) | 35 (14.58) |  |  |
| **IADL ^c^, n (%)** |  |  |  | χ²=7.65 | 0.006 |
| No | 1,074 (44.25) | 988 (45.18) | 86 (35.83) |  |  |
| Yes | 1,353 (55.75) | 1,199 (54.82) | 154 (64.17) |  |  |
| **Dietary taste, n (%)** |  |  |  | χ²=1.97 | 0.742 |
| Light taste | 1,646 (67.82) | 1,482 (67.76) | 164 (68.33) |  |  |
| Salty taste | 542 (22.33) | 484 (22.13) | 58 (24.17) |  |  |
| Sweet taste | 100 (4.12) | 92 (4.21) | 8 (3.33) |  |  |
| Hot taste | 52 (2.14) | 48 (2.19) | 4 (1.67) |  |  |
| Other | 87 (3.58) | 81 (3.70) | 6 (2.50) |  |  |
| **Hypertension, n (%)** |  |  |  | χ²=0.25 | 0.619 |
| No | 1,116 (45.98) | 1,002 (45.82) | 114 (47.50) |  |  |
| Yes | 1,311 (54.02) | 1,185 (54.18) | 126 (52.50) |  |  |
| **Diabetes, n (%)** |  |  |  | χ²=0.17 | 0.676 |
| No | 2,055 (84.67) | 1,854 (84.77) | 201 (83.75) |  |  |
| Yes | 372 (15.33) | 333 (15.23) | 39 (16.25) |  |  |
| **Heart disease, n (%)** |  |  |  | χ²=0.00 | 0.987 |
| No | 1,890 (77.87) | 1,703 (77.87) | 187 (77.92) |  |  |
| Yes | 537 (22.13) | 484 (22.13) | 53 (22.08) |  |  |
| **Stroke or CVD ^d^, n (%)** |  |  |  | χ²=0.29 | 0.589 |
| No | 2,120 (87.35) | 1,913 (87.47) | 207 (86.25) |  |  |
| Yes | 307 (12.65) | 274 (12.53) | 33 (13.75) |  |  |
| **Self-reported quality of life, n (%**) |  |  |  | χ²=104.47 | <.001 |
| Very good | 704 (29.01) | 668 (30.54) | 36 (15.00) |  |  |
| Good | 1,129 (46.52) | 1,037 (47.42) | 92 (38.33) |  |  |
| So so | 545 (22.46) | 453 (20.71) | 92 (38.33) |  |  |
| Bad | 41 (1.69) | 24 (1.10) | 17 (7.08) |  |  |
| Very bad | 8 (0.33) | 5 (0.23) | 3 (1.25) |  |  |
| **Self-reported health status, n (%)** |  |  |  | χ²=100.45 | <.001 |
| Very good | 367 (15.12) | 361 (16.51) | 6 (2.50) |  |  |
| Good | 916 (37.74) | 855 (39.09) | 61 (25.42) |  |  |
| So so | 859 (35.39) | 751 (34.34) | 108 (45.00) |  |  |
| Bad | 269 (11.08) | 209 (9.56) | 60 (25.00) |  |  |
| Very bad | 16 (0.66) | 11 (0.50) | 5 (2.08) |  |  |
| **Age, n (%)** |  |  |  | χ²=6.76 | 0.080 |
| 65-70 | 513 (21.14) | 451 (20.62) | 62 (25.83) |  |  |
| 71-80 | 809 (33.33) | 723 (33.06) | 86 (35.83) |  |  |
| 81-90 | 621 (25.59) | 566 (25.88) | 55 (22.92) |  |  |
| >90 | 484 (19.94) | 447 (20.44) | 37 (15.42) |  |  |
| **Looking on the bright side, n (%)** |  |  |  | χ²=348.95 | <.001 |
| Always/Often | 81 (3.34) | 35 (1.60) | 46 (19.17) |  |  |
| Sometimes | 310 (12.77) | 226 (10.33) | 84 (35.00) |  |  |
| Seldom/Never | 2,036 (83.89) | 1,926 (88.07) | 110 (45.83) |  |  |
| **Keeping tidy and clean, n (%)** |  |  |  | χ²=39.47 | <.001 |
| Always/Often | 55 (2.27) | 40 (1.83) | 15 (6.25) |  |  |
| Sometimes | 669 (27.56) | 576 (26.34) | 93 (38.75) |  |  |
| Seldom/Never | 1,703 (70.17) | 1,571 (71.83) | 132 (55.00) |  |  |
| **Feeling energetic, n (%)** |  |  |  | χ²=78.83 | <.001 |
| Always/Often | 370 (15.25) | 295 (13.49) | 75 (31.25) |  |  |
| Sometimes | 658 (27.11) | 573 (26.20) | 85 (35.42) |  |  |
| Seldom/Never | 1,399 (57.64) | 1,319 (60.31) | 80 (33.33) |  |  |
| **Feeling ashamed/regretful/guilty, n (%)** |  |  |  | χ²=105.70 | <.001 |
| Always/Often | 1,972 (81.25) | 1,836 (83.95) | 136 (56.67) |  |  |
| Sometimes | 373 (15.37) | 288 (13.17) | 85 (35.42) |  |  |
| Seldom/Never | 82 (3.38) | 63 (2.88) | 19 (7.92) |  |  |
| **Feeling angry, n (%)** |  |  |  | χ²=106.93 | <.001 |
| Always/Often | 1,966 (81.01) | 1,829 (83.63) | 137 (57.08) |  |  |
| Sometimes | 356 (14.67) | 284 (12.99) | 72 (30.00) |  |  |
| Seldom/Never | 105 (4.33) | 74 (3.38) | 31 (12.92) |  |  |
| **Feeling busy, n (%)** |  |  |  | χ²=19.72 | <.001 |
| Almost/At least once a week | 266 (10.96) | 230 (10.52) | 36 (15.00) |  |  |
| At least once a month | 367 (15.12) | 312 (14.27) | 55 (22.92) |  |  |
| Not every month/never | 1,794 (73.92) | 1,645 (75.22) | 149 (62.08) |  |  |
| **Feeling people not trustworthy, n (%)** |  |  |  | χ²=81.19 | <.001 |
| Always/Often | 1,834 (75.57) | 1,696 (77.55) | 138 (57.50) |  |  |
| Sometimes | 234 (9.64) | 173 (7.91) | 61 (25.42) |  |  |
| Seldom/Never | 359 (14.79) | 318 (14.54) | 41 (17.08) |  |  |
| **Staple food, n (%)** |  |  |  | χ²=11.17 | 0.025 |
| Rice | 1,215 (50.06) | 1,077 (49.25) | 138 (57.50) |  |  |
| Corn | 111 (4.57) | 96 (4.39) | 15 (6.25) |  |  |
| Wheat | 503 (20.73) | 457 (20.90) | 46 (19.17) |  |  |
| Half rice and half wheat | 577 (23.77) | 537 (24.55) | 40 (16.67) |  |  |
| Other | 21 (0.87) | 20 (0.91) | 1 (0.42) |  |  |
| **Fresh fruits, n (%)** |  |  |  | χ²=42.76 | <.001 |
| Everyday/Almost everyday | 717 (29.54) | 678 (31.00) | 39 (16.25) |  |  |
| Quite often | 571 (23.53) | 529 (24.19) | 42 (17.50) |  |  |
| Occasionally | 639 (26.33) | 556 (25.42) | 83 (34.58) |  |  |
| Rarely/Never | 500 (20.60) | 424 (19.39) | 76 (31.67) |  |  |
| **Fresh vegetables, n (%)** |  |  |  | χ²=24.88 | <.001 |
| Everyday/Almost everyday | 1,751 (72.15) | 1,606 (73.43) | 145 (60.42) |  |  |
| Quite often | 503 (20.73) | 441 (20.16) | 62 (25.83) |  |  |
| Occasionally | 123 (5.07) | 100 (4.57) | 23 (9.58) |  |  |
| Rarely/Never | 50 (2.06) | 40 (1.83) | 10 (4.17) |  |  |
| **Cooking oil, n (%)** |  |  |  | χ²=5.99 | 0.014 |
| Vegetable oil/gingili oil | 2,273 (93.65) | 2,057 (94.06) | 216 (90.00) |  |  |
| Animal fat | 154 (6.35) | 130 (5.94) | 24 (10.00) |  |  |
| **Housework, n (%)** |  |  |  | χ²=2.11 | 0.348 |
| Almost/At least once a week | 952 (39.23) | 868 (39.69) | 84 (35.00) |  |  |
| At least once a month | 192 (7.91) | 173 (7.91) | 19 (7.92) |  |  |
| Sometimes/Never | 1,283 (52.86) | 1,146 (52.40) | 137 (57.08) |  |  |
| **Taichi chuan, n (%)** |  |  |  | - | 0.298 |
| Almost/At least once a week | 2,359 (97.20) | 2,126 (97.21) | 233 (97.08) |  |  |
| At least once a month | 22 (0.91) | 18 (0.82) | 4 (1.67) |  |  |
| Sometimes/Never | 46 (1.90) | 43 (1.97) | 3 (1.25) |  |  |
| **Square dance, n (%)** |  |  |  | χ²=2.81 | 0.245 |
| Almost/At least once a week | 2,302 (94.85) | 2,074 (94.83) | 228 (95.00) |  |  |
| At least once a month | 45 (1.85) | 38 (1.74) | 7 (2.92) |  |  |
| Sometimes/Never | 80 (3.30) | 75 (3.43) | 5 (2.08) |  |  |
| **Interaction with friends, n (%)** |  |  |  | χ²=5.75 | 0.056 |
| Almost/At least once a week | 1,068 (44.00) | 950 (43.44) | 118 (49.17) |  |  |
| At least once a month | 566 (23.32) | 506 (23.14) | 60 (25.00) |  |  |
| Sometimes/Never | 793 (32.67) | 731 (33.42) | 62 (25.83) |  |  |
| **Other outdoor activities, n (%)** |  |  |  | χ²=5.62 | 0.060 |
| Almost/At least once a week | 1,637 (67.45) | 1,472 (67.31) | 165 (68.75) |  |  |
| At least once a month | 271 (11.17) | 236 (10.79) | 35 (14.58) |  |  |
| Sometimes/Never | 519 (21.38) | 479 (21.90) | 40 (16.67) |  |  |
| **Garden work, n (%)** |  |  |  | χ²=1.92 | 0.384 |
| Almost/At least once a week | 1,868 (76.97) | 1,676 (76.63) | 192 (80.00) |  |  |
| At least once a month | 105 (4.33) | 98 (4.48) | 7 (2.92) |  |  |
| Sometimes/Never | 454 (18.71) | 413 (18.88) | 41 (17.08) |  |  |
| **Reading books or newspapers, n (%)** |  |  |  | χ²=11.89 | 0.003 |
| Almost/At least once a week | 1,838 (75.73) | 1,636 (74.81) | 202 (84.17) |  |  |
| At least once a month | 197 (8.12) | 180 (8.23) | 17 (7.08) |  |  |
| Sometimes/Never | 392 (16.15) | 371 (16.96) | 21 (8.75) |  |  |
| **Raising domestic animals, n (%)** |  |  |  | χ²=9.71 | 0.008 |
| Almost/At least once a week | 1,944 (80.10) | 1,770 (80.93) | 174 (72.50) |  |  |
| At least once a month | 70 (2.88) | 61 (2.79) | 9 (3.75) |  |  |
| Sometimes/Never | 413 (17.02) | 356 (16.28) | 57 (23.75) |  |  |
| **Playing cards or mahjong, n (%)** |  |  |  | χ²=6.12 | 0.047 |
| Almost/At least once a week | 1,983 (81.71) | 1,775 (81.16) | 208 (86.67) |  |  |
| At least once a month | 220 (9.06) | 200 (9.14) | 20 (8.33) |  |  |
| Sometimes/Never | 224 (9.23) | 212 (9.69) | 12 (5.00) |  |  |
| **Watching TV or listening to the radio, n (%)** |  |  |  | χ²=23.76 | <.001 |
| Almost/At least once a week | 435 (17.92) | 367 (16.78) | 68 (28.33) |  |  |
| At least once a month | 284 (11.70) | 250 (11.43) | 34 (14.17) |  |  |
| Sometimes/Never | 1,708 (70.37) | 1,570 (71.79) | 138 (57.50) |  |  |
| **Social activities, n (%)** |  |  |  | χ²=0.91 | 0.634 |
| Almost/At least once a week | 2,148 (88.50) | 1,932 (88.34) | 216 (90.00) |  |  |
| At least once a month | 194 (7.99) | 176 (8.05) | 18 (7.50) |  |  |
| Sometimes/Never | 85 (3.50) | 79 (3.61) | 6 (2.50) |  |  |
| **Traveling times, n (%)** |  |  |  | χ²=3.01 | 0.222 |
| 0 | 2,006 (82.65) | 1,798 (82.21) | 208 (86.67) |  |  |
| 1 | 136 (5.60) | 126 (5.76) | 10 (4.17) |  |  |
| ≥2 | 285 (11.74) | 263 (12.03) | 22 (9.17) |  |  |

Note:χ²: Chi-square test, -: Fisher exact,P: p-value

a:BMI indicates Body Mass Index

b:ADL indicates Activity of Daily Living

c:IADL indicates Instrumental Activity of Daily Living

d:CVD indicates Cerebrovascular disease
